# Supplementary material for: Soil microbial community response to corrinoids is shaped by a natural reservoir of vitamin B12
Source: ISME J. 2024 Jun 4;18(1):wrae094. doi: 10.1093/ismejo/wrae094 (PMC11287211; doi:10.1093/ismejo/wrae094)
Supplement: 20240426_Soil_Supplemental_Figures_wrae094 [file 20240426_soil_supplemental_figures_wrae094.pdf]

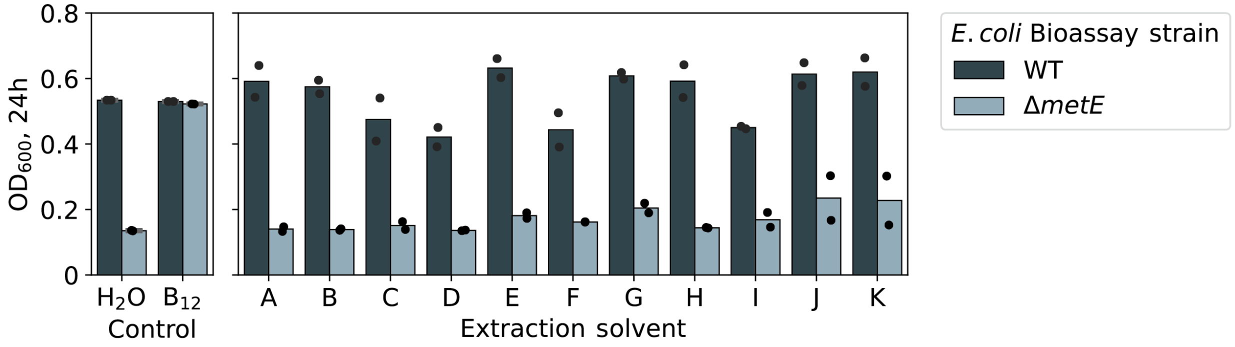

Supplemental Figure 1. Quantification of total corrinoid in organic extractions from soil by microbiological assay. Growth of the control *E. coli* MG1655 WT or corrinoid-responsive *ΔmetE* strains in M9-glycerol (0.4%) with added extract from soil obtained using the indicated organic solvent conditions. Solution A - MeOH:H_2O (4:1), Solution B - $CHCl_3$:MeOH (2:1), Solution C - EtOAc, Solution D - Acidic MeOH:H_2O (4:1), Solution E - Basic MeOH:H_2O (4:1), Solution F - Acidic MeCN:MeOH:H_2O (2:2:1), Solution G - Basic MeCN:MeOH:H_2O (2:2:1), Solution H - MeCN:MeOH:H_2O (2:2:1), Solution I - Acidic MeCN:H_2O (4:1), Solution J - Basic MeCN:H_2O (4:1), Solution K - MeCN:H_2O (4:1). Acidic extraction solutions included 0.1 M formic acid, and basic solutions included 0.1 M ammonium hydroxide. CN-Cbl (vitamin B_{12}, 1 nM) or water (H_2O) were added as controls.

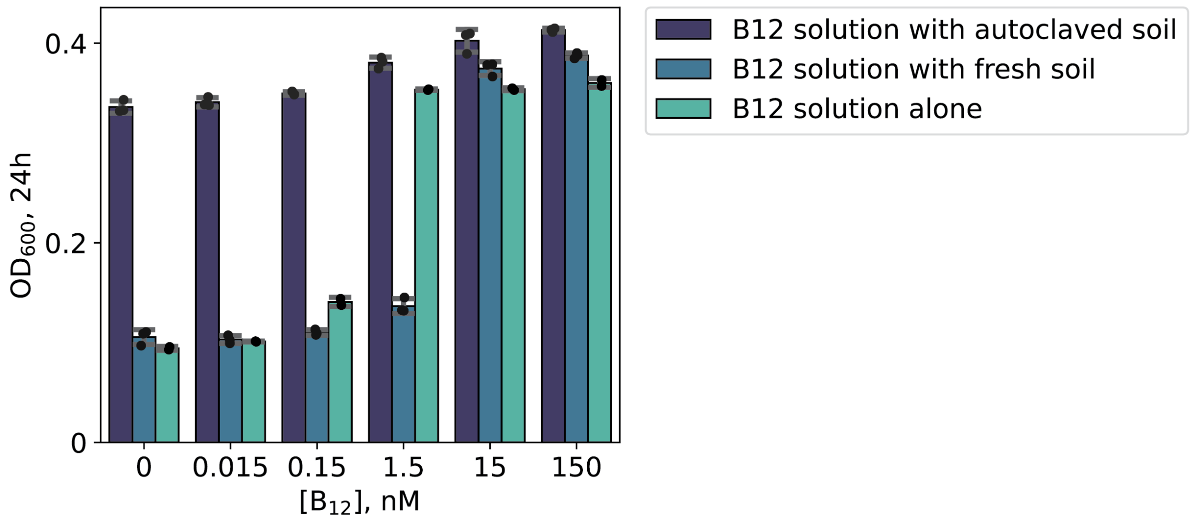

Supplemental Figure 2. B_{12} adsorbs to soil. Growth of the corrinoid-responsive *E. coli ΔmetE* strain in M9-glycerol (0.4%) supplemented with CN-Cbl (vitamin B_{12}) solutions at the indicated concentrations pre-incubated with or without fresh or autoclaved soil.

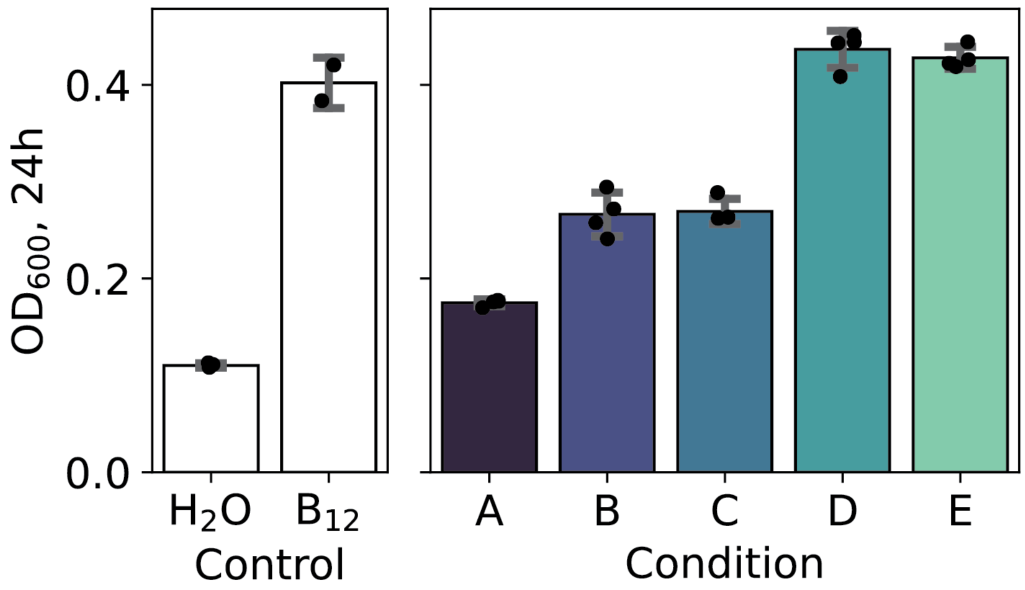

Supplemental Figure 3. Effect of steam sterilization and phosphate buffer on corrinoid extraction from soil. Growth of the corrinoid-responsive *E. coli ΔmetE* strain supplemented with control solutions of CN-Cbl (B_{12}, 1 nM) or water (H_2O), or extracts from soil: A - Fresh soil extracted with water, B - Fresh soil extracted with 0.1 M potassium phosphate (pH 7.0) solution, C - Autoclaved soil extracted with water, D - Autoclaved soil extracted with phosphate solution, E - Autoclaved soil that was moistened immediately before sterilization.

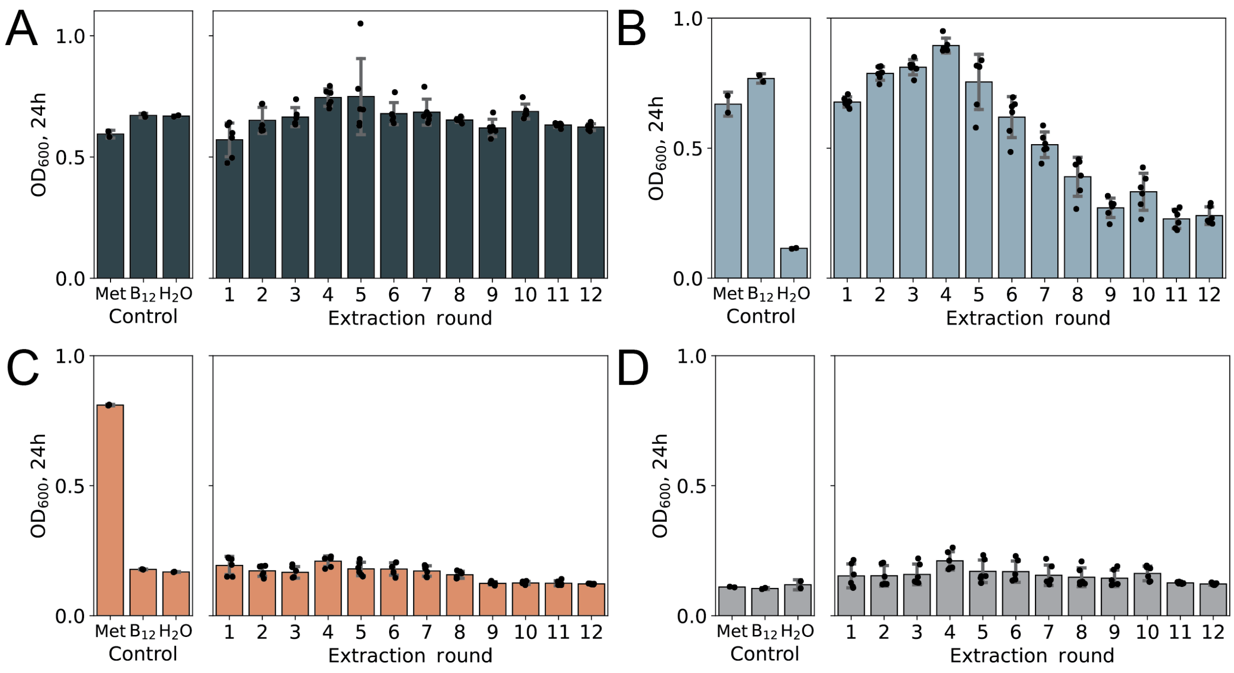

Supplemental Figure 4. Controls for corrinoid quantification bioassay. OD_{600} measurements of *E. coli* strains: (A) WT, (B) *ΔmetE*, (C) *ΔmetEΔmetH*, and (D) uninoculated control in serial extraction rounds of soil using M9-glycerol (0.4%), alongside controls supplemented with methionine (Met, 6.7 mM), CN-Cbl (B_{12}, 1 nM), or water (H_2O). Panel B is a reproduction of Figure 3A, included here for readability.

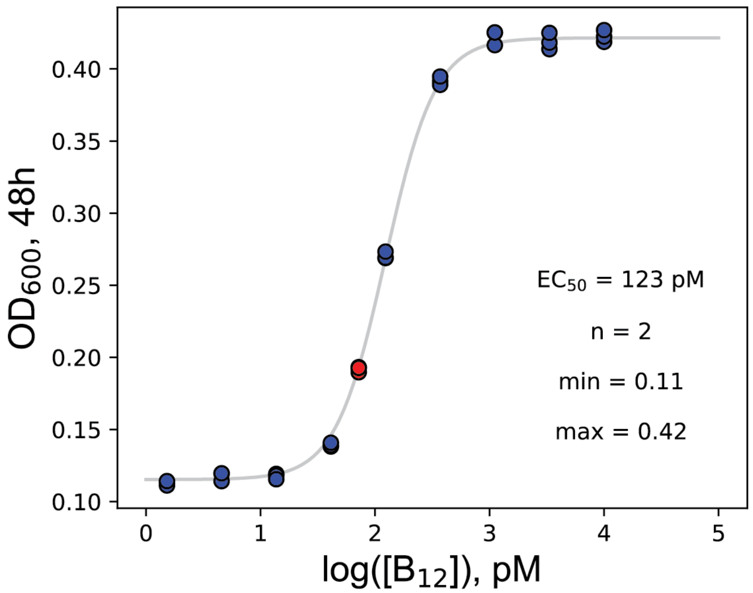

Supplemental Figure 5.Quantification of total corrinoid in soil extracts in BtuG-depleted soil. Dose-response curve of *E. coli ΔmetE* against CN-Cbl (B_{12}) in corrinoid-depleted soil extract (Blue), and corresponding measurement in control soil extract not depleted for cobamide by BtuG (Red). Soil extract used for this experiment was obtained by combining all 8 rounds of M9-based extractions (Figure 3A). Corrinoid-depleted soil extract was obtained by incubation with the high-affinity corrinoid-binding protein BtuG, followed by protein removal using Ni-NTA agarose beads. The control soil extract was treated only with Ni-NTA agarose beads.

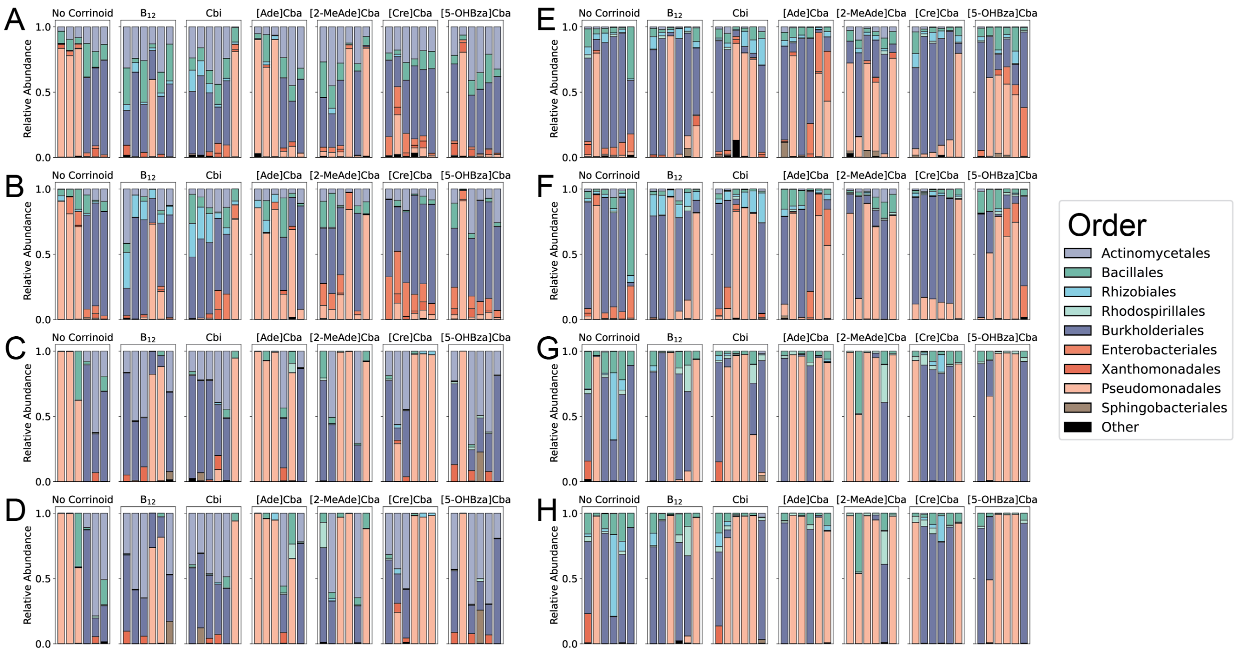

Supplemental Figure 6. Relative abundances of taxa in soil-derived enrichment cultures based on 16S rRNA amplicon sequencing. Relative abundances at the order level of enrichments in M9 (A-D) and R2 (E-H), grouped by corrinoid condition at (A,E) 1 week, (B,F) 2 weeks, (C,G) 12 weeks, and (D,H) 14 weeks. Six replicates are shown for each condition.

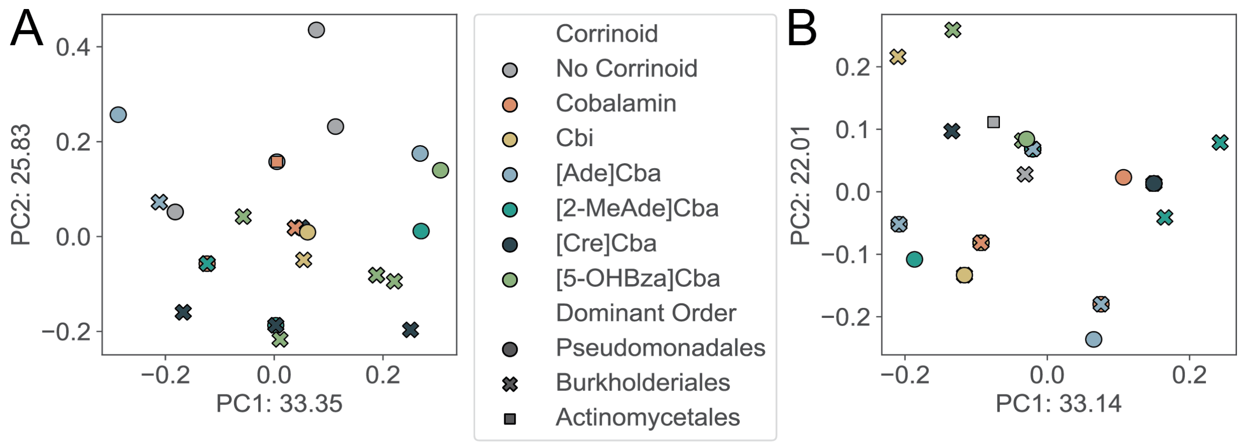

Supplemental Figure 7. PCoA analysis using Jaccard dissimilarity metric of soil-derived enrichment cultures at 2 weeks. A, B. Principal coordinate analysis (PCoA) plot with Jaccard dissimilarity of M9 (A) and R2 (B) enrichments at the order level grouped by corrinoid condition at 2 weeks. M9 enrichments (A) significantly cluster by corrinoid (ANOSIM: r = 0.1189, P < 0.05, PERMANOVA: r = 2.407, P < 0.05). R2 enrichment cultures (B) do not cluster by corrinoid (ANOSIM: r = 0.3133, P < 0.05).

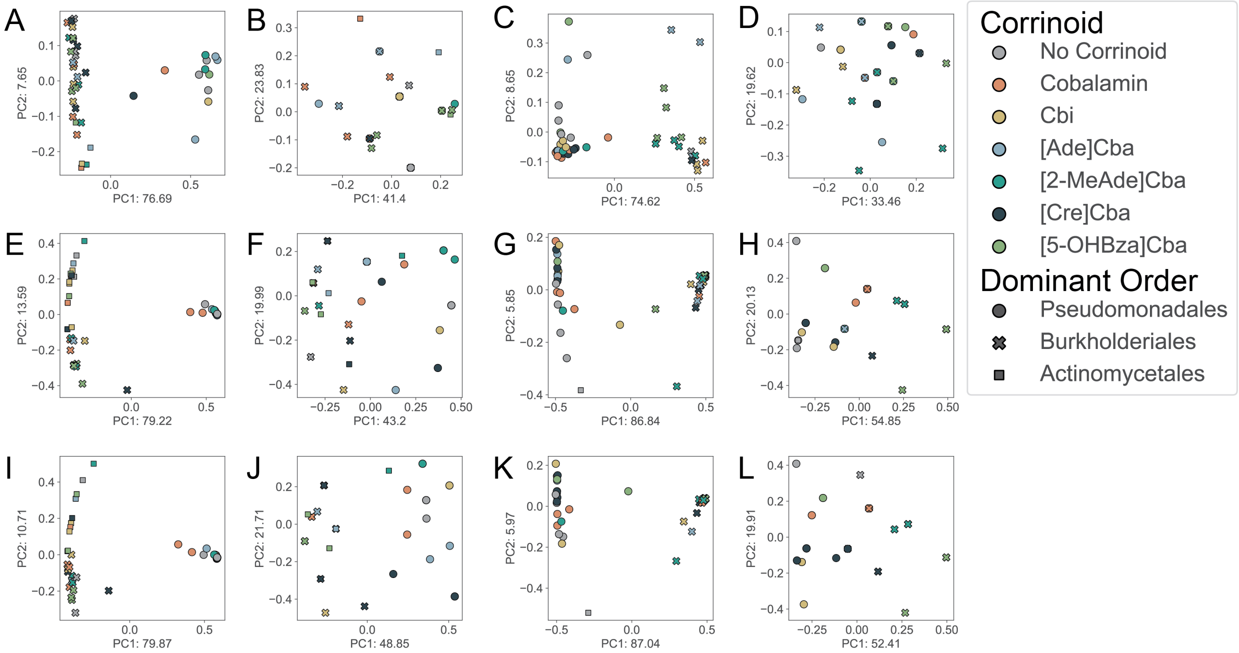

Supplemental Figure 8. PCoA analysis of soil-derived enrichment cultures at 1, 12, and 14 weeks. A-L. Principal coordinate analysis (PCoA) plot with Bray-Curtis (A,C,E,G,I,K) and Jaccard (B,D,F,H,J,L) dissimilarity of M9 (A,B,E,F,I,J) and R2 (C,D,G,H,K,L) enrichments at the order level at 1 (A-D), 12 (E-H), and 14 (I-L) weeks. No significant clustering is observed at these time points (ANOSIM/PERMANOVA, $P > 0.05$).

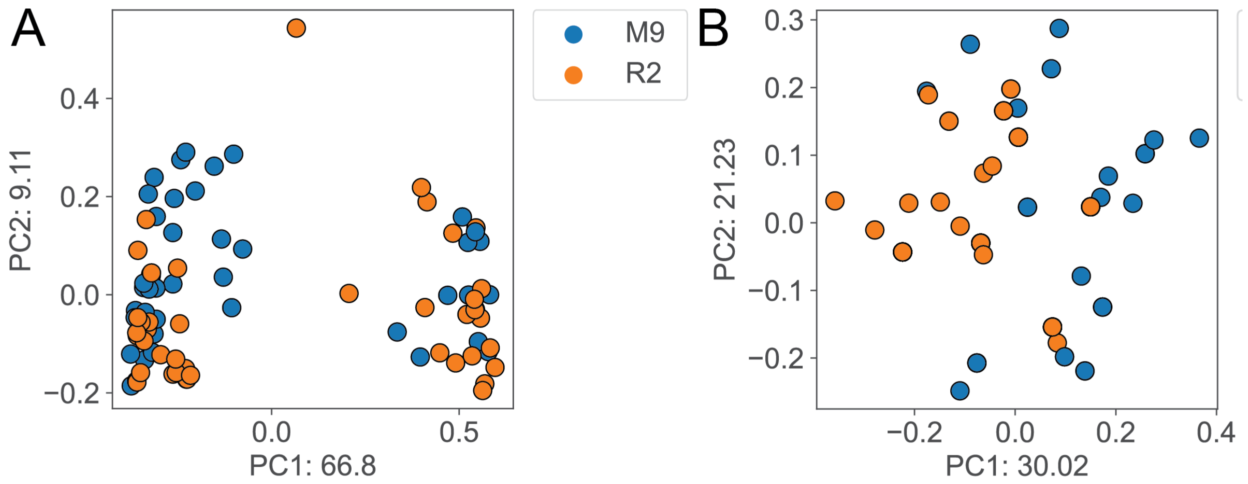

Supplemental Figure 9. Clustering of soil-derived enrichments by culture medium. A, B. Principal coordinate analysis (PCoA) plot with Bray-Curtis (A) and Jaccard (B) dissimilarity of M9 and R2 enrichments at the order level, grouped by medium (M9 and R2) at 2 weeks. Enrichments significantly cluster by medium using both (A) Bray-Curtis (ANOSIM: $r = 0.0652$, $P < 0.05$; PERMANOVA: $r = 3.8217$, $P < 0.05$) and (B) Jaccard (ANOSIM: $r = 0.3459$, $P < 0.05$; PERMANOVA: $r = 24.3267$, $P < 0.05$) metrics.

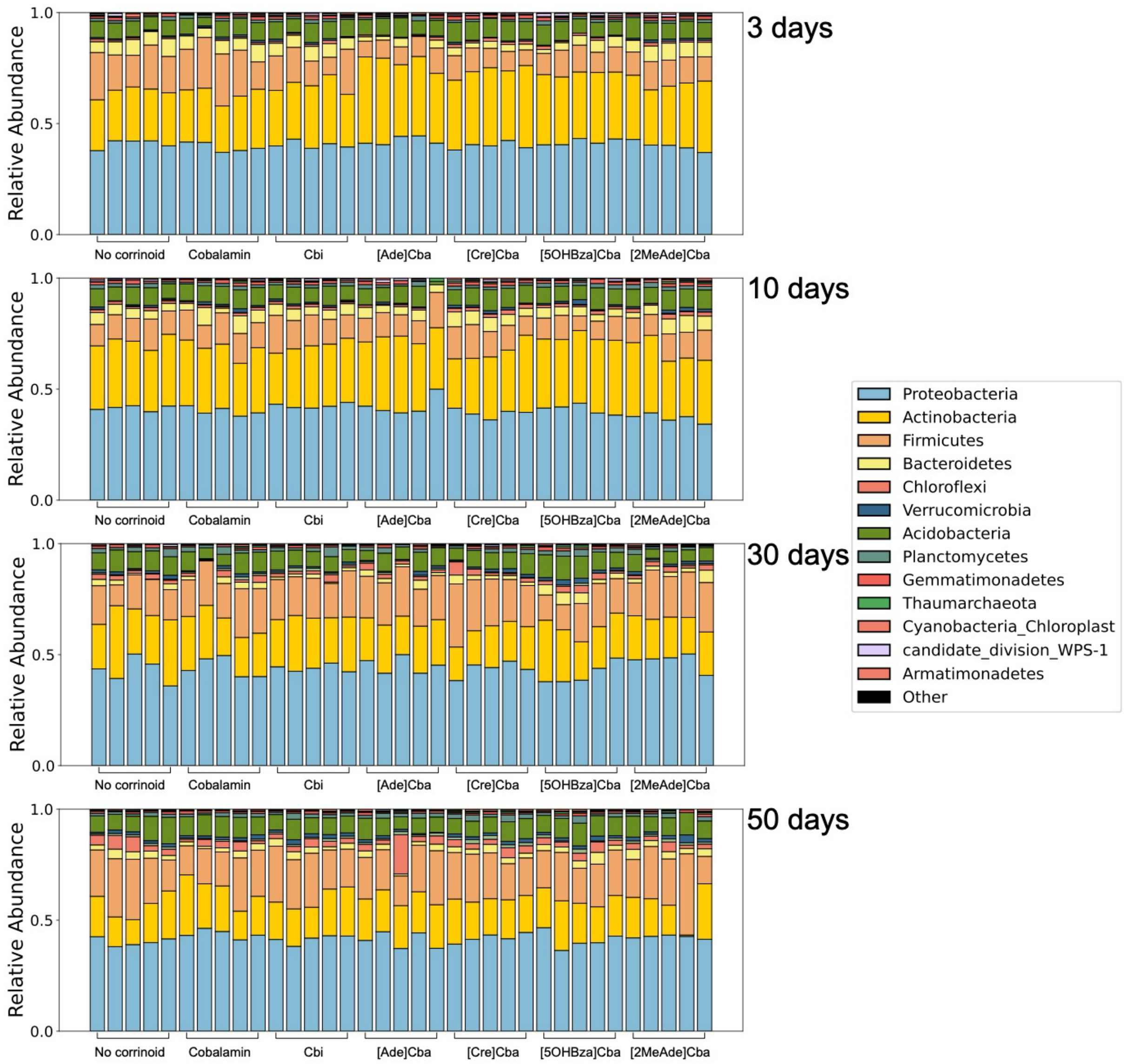

Supplemental Figure 10. Composition of soil microcosms through time. Stacked bar charts of the top ten phyla represented by colors, grouped by corrinoid treatment. Graphs are arranged through time where the top plot shows day 3 and the bottom plot shows day 50. Phyla are arranged with the most prevalent on the bottom and the least prevalent on the top.

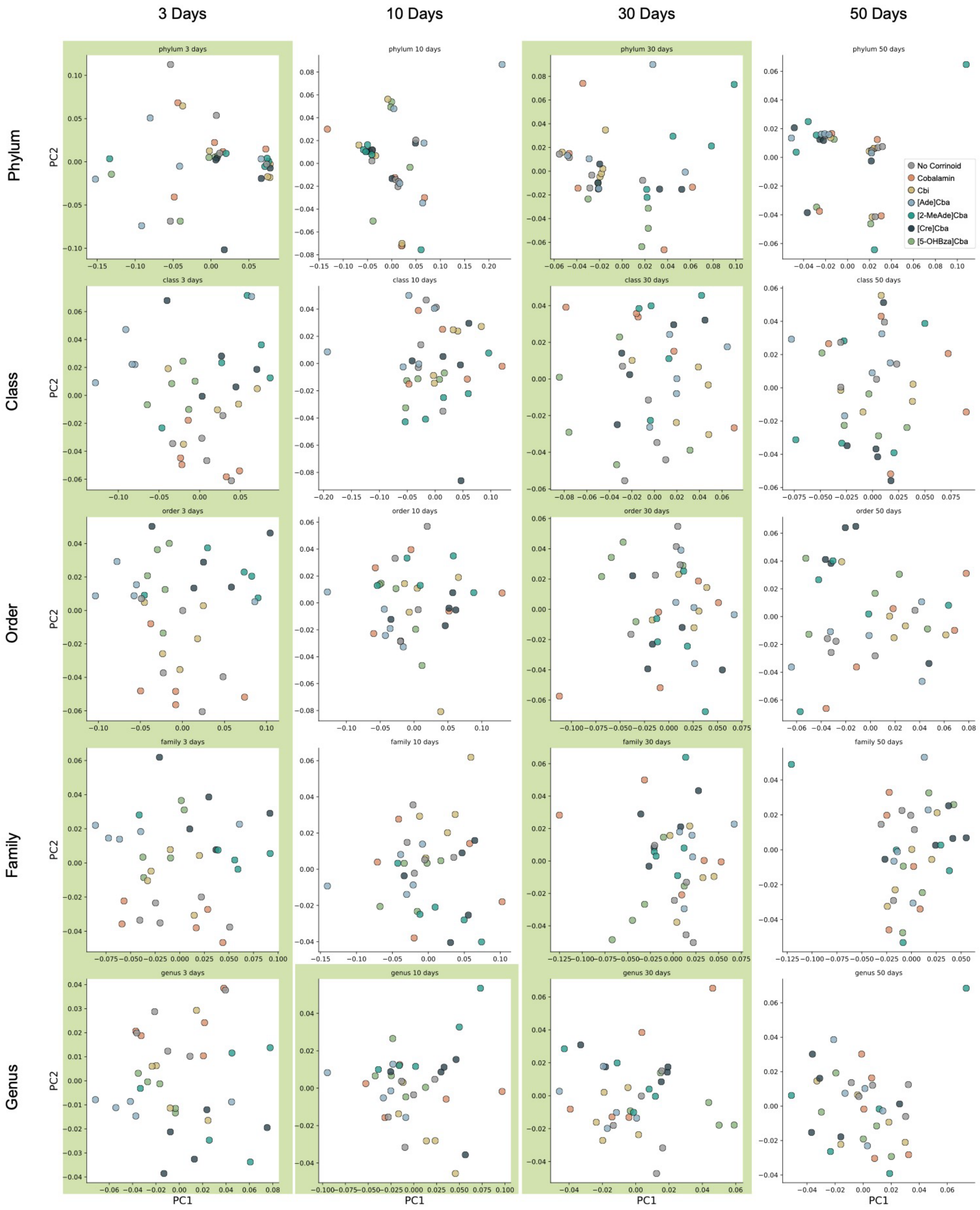

Supplemental Figure 11. Corrinoid treatment effects on soil microcosms. PCoA of Bray-Curtis dissimilarity aggregated by taxonomy (top: phylum, bottom: genus) through time (left: day 3, right: day 50). Each plot shows the first two principal components with the percent variance explained. Green shading indicates statistical significance (ANOSIM and PERMANOVA, $P \leq 0.05$).
